# Heavy chain deposition disease presenting with raised anti-GBM antibody levels; a case report

**DOI:** 10.1186/s12882-020-01837-2

**Published:** 2020-05-12

**Authors:** Michael Turner, Anna Crawford, Claire Winterbottom, Oliver Flossmann, Bassam Alchi, Maria Soares, Umanath Bhandary

**Affiliations:** 1 Renal Unit, Royal Berkshire Hospital, London Road, Reading, Berkshire, UK; 2 Department of Cellular Pathology, John Radcliffe Hospital, Headley Way, Oxford, UK

**Keywords:** Monoclonal Immunogloblin deposition disease, Myeloma, Anti-GBM, Case report

## Abstract

**Background:**

Monoclonal immunoglobulin deposition disease (MIDD) is a rare condition accounting for < 1% of histopathological diagnoses made on kidney biopsy^1^. The best outcomes are seen in those diagnosed and treated promptly, but delay to diagnosis is common with the largest series reporting a median time from onset of renal impairment to diagnosis of 12 months^2^. Here, we report a case of the heavy chain subset of MIDD presenting with positive anti-glomerular basement membrane (anti-GBM) antibodies obscuring the true diagnosis.

**Case presentation:**

Here, we present a challenging case presenting with oedema, haematoproteiuria, and new renal impairment. Anti-GBM antibodies were positive and prompted treatment as atypical anti-GBM disease. However, they were ultimately proven to be monoclonal and secondary to myeloma. The final diagnosis facilitated effective myeloma treatment which led to complete remission and independence from renal replacement therapy.

**Conclusions:**

This case reinforces the importance of comprehensive histopathological and haematological assessment in making the correct diagnosis. Here it facilitated effective treatment and recovery of renal function.

## Background

Monoclonal immunoglobulin deposition disease (MIDD) is a rare condition accounting for < 1% of histopathological diagnoses made on kidney biopsy [1]. Deposition of monoclonal immunoglobulin proteins (light chains, heavy chains, or both) within the basement membranes leads to progressive renal impairment. Prompt treatment of the underlying plasma cell disorder offers the best chances of good results. However, delay in diagnosis is frequent, with median time from onset to diagnosis being 1 year in a large series [2].

Anti-glomerular basement membrane (GBM) disease is caused by antibodies targeted against the non-collagenous (NC1) domain of the a3 chain of type IV collagen (a3[IV]NC1c) [3]. Atypical presentations with haematoproteinuria and less rapid deterioration in renal function are well-described [3]. Anti-GBM antibodies are detectable in patient serum and are often considered diagnostic. However, false positives and negatives have been described [3, 4]. Histopathological confirmation offers greater certainty in the diagnosis of anti-GBM disease and may be sought through observation of linear IgG deposition in the basement membrane on kidney biopsy [4]. Here we report a case presenting with haematoproteinuria, renal impairment, circulating anti-GBM antibodies, and linear IgG deposition in the glomerular basement membranes. However, they ultimately proved to have heavy chain deposition disease (HCDD). Myeloma treatment led to abrogation of antibody production and a good clinical outcome.

## Case presentation

A previously fit and well 48 year-old Caucasian male, with no significant past medical history, presented with a 3 month history of foot swelling. He reported no other symptoms. Physical examination demonstrated oedema to the knees, but no other findings of note. Urine dipstick showed blood +++ and protein +++.

He had impaired renal function with a creatinine of 186micromol/L, corresponding to an eGFR of 34 mL/min/1.73m^2^. CRP was 4 mg/L, albumin 27 g/L and Hb 113 g/L. His urine protein:creatinine ratio was 228.4 mg/mmol. An immunology screen showed a raised anti-GBM level of 32 units/mL. Anti-neutrophil cytoplasmic antibody (ANCA) and anti-nuclear antibodies (ANA) were both negative. Serum protein electrophoresis showed a gamma paraprotein which was too small to quantify, and an elevated kappa band at 182 mg/L with a normal lambda band of 16.80 mg/L (ratio: 10.83). C3 and C4 levels were normal and a virology screen was negative for HIV, hepatitis B virus and hepatitis C virus. On computed tomography, there was neither evidence of pulmonary haemorrhage nor any lymphadenopathy within the neck, chest, abdomen or pelvis. Although light chains were noted, their raised values were interpreted as a result of renal impairment generally and atypical anti-GBM disease specifically [3]. Therefore, clinical concern regarding atypical anti-GBM disease led to commencement of steroids, cyclophosphamide and plasma exchange. A biopsy was performed for histopathological confirmation.

**Fig. 1:**
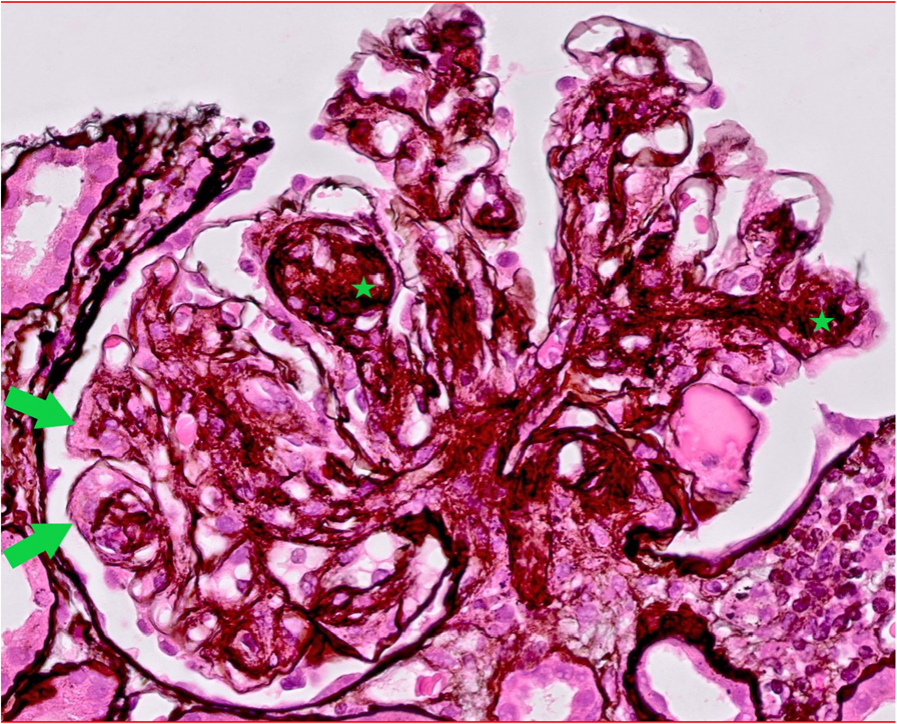
Glomeruli show diffuse mesangial expansion with nodule formation (*) which are positive on silver stain (silver stain 400x). There is focal basement membrane duplication (arrows)

Light microscopy showed ten glomeruli, with none being globally sclerosed. There was mixed nodular sclerosis with focal mesangial and endocapillary hypercellularity. Focal basement membrane duplication was seen on silver stain (Fig. 1). There was no necrosis and no crescents. There was mild chronic damage with 10% interstitial fibrosis and tubular atrophy. Due to the need to transport biological samples between centres, detection of immunoglobulins, complement and light chain fractions was performed by immunoperoxidase staining on formalin-fixed paraffin-embedded tissue. This showed linear glomerular and tubular basement membrane positivity for IgG. IgA and IgM were negative. C3 and C1q showed mesangial positivity in the sclerosing lesions. Kappa and lambda staining was negative in the glomeruli. Immunohistochemical and light microscopy features were felt to be in keeping with atypical anti-GBM disease, but the possibility of a monoclonal immunoglobulin deposition disease was considered in the differential diagnosis. Therefore electron microscopy was imperative.

**Fig. 2:**
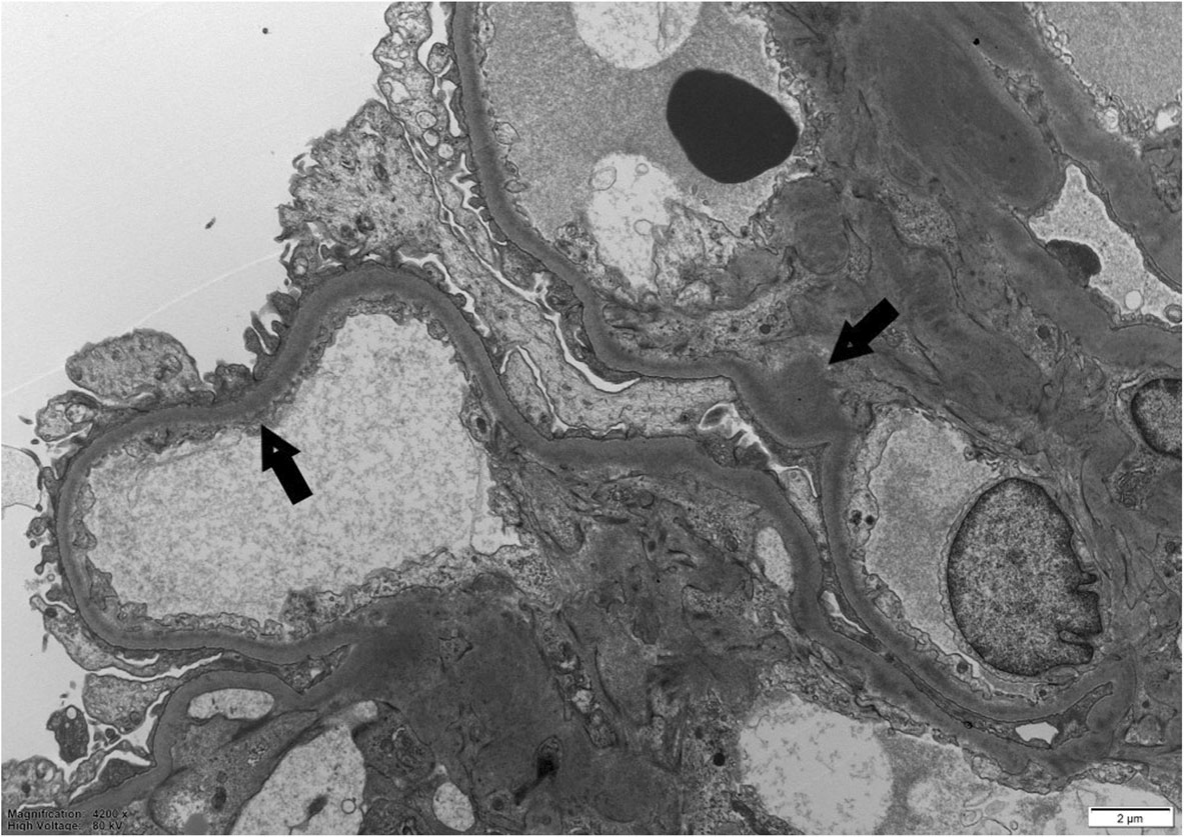
Transmission electron microscopy shows powdery electron dense deposits along basement membranes (× 4200)

The GBM level peaked at 43 units/mL and showed a limited response to plasma exchange, persisting at 25 units/mL. The gentleman’s creatinine deteriorated to 445micromol/L during this time. Electron microscopy demonstrated powdery electron dense deposits along the lamina rara interna of glomerular basement membranes (Fig. 2) and also along tubular basement membranes, in keeping with monoclonal immunoglobulin deposition disease. The Haematologists were consulted and a bone marrow biopsy showed a 10–20% infiltration by kappa-restricted plasma cells. A del 17 P clone was detected on plasma cell fluorescent in-situ hybridisation. He was started on bortezemib and dexamethasone and became haemodialysis-dependent for 4 months. Daratumumab was added to his myeloma treatment regimen and he achieved a complete remission. He subsequently had a stem cell transplant. His anti-GBM titre is now undetectable and his renal function stable with a creatinine of 108micromol/L corresponding to an eGFR of 69 mL/min/1.73m^2^.

## Discussion and conclusions

This gentleman presented with haematoproteinuria and renal impairment. His serum contained circulating anti-GBM antibodies and histopathology showed linear IgG deposition along the basement membrane, although we were not able to demonstrate polyclonality in keeping with an autoimmune reaction. The patient was initially treated as atypical anti-GBM disease with steroids, cyclophosphamide and plasma exchange. Atypical cases of anti-GBM disease presenting with milder renal impairment and significant levels of proteinuria have been well-described and treatment as in classical anti-GBM disease produces favourable results [5, 6]. Treating as anti-GBM disease here proved ineffective and adequate treatment was delayed for weeks until electron microscopy results were available. Haematological work-up unearthed myeloma and guaranteed first-line treatment which here has been successful in achieving remission and dialysis independence.

Elevated anti-GBM antibodies are not commonly observed in healthy individuals. One study has reported five healthy individuals with circulating levels, but the frequency at which this occurs is not clear [7]. A report of 30 cases and 30 matched controls found 4 of the anti-GBM disease cases to have had elevated anti-GBM titres in the year prior to presentation. None of the controls had detectable anti-GBM titres [8].

False positive anti-GBM antibody assays have previously been reported [4, 9], however this patient did not have HIV or another condition previously associated with this. Additionally, on kidney biopsy this patient exhibited linear GBM IgG positivity. The disappearance of this patient’s antibody following successful treatment is in keeping with a myeloma paraprotein with affinity to the anti-GBM antibody assay. Immunoglobulins produced by plasma cell dyscrasias have been demonstrated to possess reactivity to recognised auto- and xeno-antigens. In one series, the sera of 75 patients with monoclonal gammopathy were examined and 17 (23%) reacted to a panel including DNA, extractable nuclear antigens and cardiolipin [10]. Another series found that 10 of 46 patients with multiple myeloma had sera which demonstrated reactivity to mycobacterial glycolipids. 2 of these 10 were selected for further testing and reacted to nuclear antigens [11]. In patients with unusual presentations, who are not responding to active therapy, reconsidering of the origin and clinical significance of auto-antibodies may facilitate fruitful further investigations.

Although the immunoglobulin did not cause anti-GBM disease, it did deposit and cause a heavy chain deposition disease (HCDD). HCDD is caused by monoclonal heavy chains depositing in the glomeruli and tubules. The cationic properties of heavy chains are thought to predispose to deposition in the anionic renal basement membranes [12]. This leads to haematoproteinuria and progressive renal impairment. C1q and C3 staining is commonly positive in HCDD [12, 13]. Treatment of the underlying plasma cell dyscrasia is key to achieving a good renal outcome [13, 14]. In keeping with many rare diseases [15], delay in diagnosis occurs with MIDD with a median time to diagnosis of 12 months in a large case series [2]. Baseline eGFR < 20 mL/min/1.73m^2^ is predictive of need for renal replacement therapy [2], highlighting the importance of diagnosis early in the clinical course. Our diagnosis hinged upon electron microscopy, which took several weeks to come back. Once the final diagnosis was made, myeloma resulted in complete haematological remission followed by a good renal response. This is keeping with experience elsewhere [2, 13, 14].

Interestingly, the opposite of this situation has been described. Coley et al. described an indolent anti-GBM disease caused by a low-titre IgGκ paraprotein that evaded detection either on anti-GBM assay or via serum electrophoresis [16]. That case responded to steroids and cyclophosphamide, whereas this case did not respond to that therapy.

A strength of this case report is the comprehensive work-up with serum consistently containing circulating anti-GBM antibodies and histopathological assessment showing linear IgG deposition along the basement membrane. We are not aware of previous reports where a paraprotein caused a similar uncertainty as to the aetiology of progressive renal impairment. The chief weakness is that we were not able to demonstrate monoclonality due to the use of immunoperoxidase staining on formalin-fixed tissue. Additionally, we were not able to assess the IgG subtype of the serum paraprotein, which would have neatly demonstrated the consistency between serum and basement membrane.

Although this gentleman achieved remission from his myeloma and dialysis independence, the presence of detectable anti-GBM antibodies delayed the eventual diagnosis of myeloma. Additionally, although he did not suffer any side effects, the provision of plasma exchange did necessitate insertion of central lines and use of blood products which carry their own risks. Here, a myeloma appears to have produced an immunoglobulin which cross-reacted with the commercial anti-GBM assay. Considering the possibility of malignant, monoclonal production as opposed to auto-immune, polyclonal production was key in prompting further investigation. This case highlights the importance of thorough investigation when assessing rare conditions and the benefits of the correct diagnosis; both in terms of achieving good clinical outcomes and exposures to treatments with significant side-effects.

## Supplementary information

**Additional file 1 Fig. S1.** Clinical timeline showing events in patient’s healthcare journey.

## Data Availability

This is a case report, and data come from the electronic healthcare records of the patient. It has not been made publicly available.

## Abbreviations

ANA: Anti-nuclear antibody
ANCA: Anti-neutrophil cytoplasmic antibody
CRP: C-reactive protein
DNA: Deoxyribonucleic acid
eGFR: Estimated glomerular filtration rate
GBM: Glomerular basement membrane
HCDD: Heavy chain deposition disease
HIV: Human immunodeficiency virus
MIDD: Monoclonal immunoglobulin deposition disease
