# Supplementary material for: Heavy chain deposition disease presenting with raised anti-GBM antibody levels; a case report
Source: BMC Nephrol. 2020 May 12;21:175. doi: 10.1186/s12882-020-01837-2 (PMC7216388; doi:10.1186/s12882-020-01837-2)
Supplement: Supplementary file 1 [file 12882_2020_1837_MOESM1_ESM.docx]

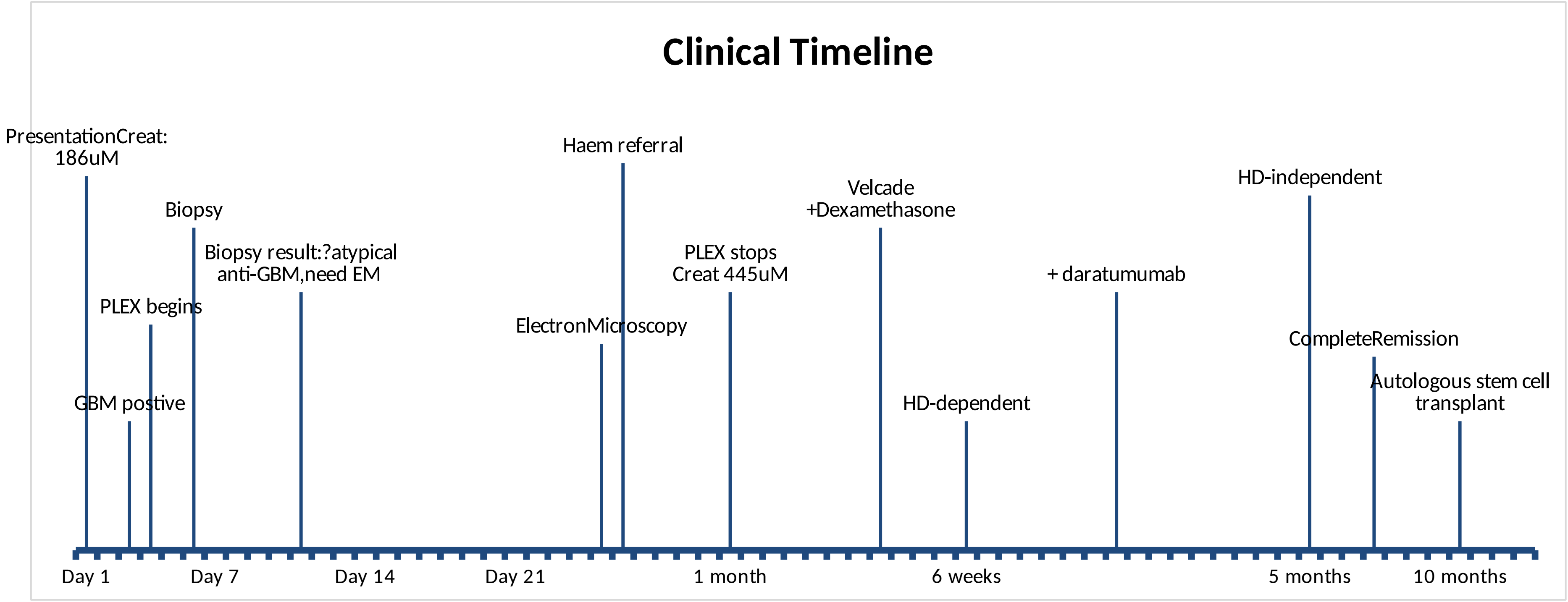
Clinical Timeline
PresentationCreat: 186uM
GBM postive
PLEX begins
Biopsy
Biopsy result:?atypical anti-GBM,need EM
ElectronMicroscopy
Haem referral
PLEX stops Creat 445uM
Velcade +Dexamethasone
HD-dependent
+ daratumumab
HD-independent
CompleteRemission
Autologous stem cell transplant
Day 1
Day 7
Day 14
Day 21
1 month
6 weeks
5 months
10 months
